# NIST Ionization Chamber “A” Sample-Height Corrections

**DOI:** 10.6028/jres.117.003

**Published:** 2012-02-02

**Authors:** Ryan Fitzgerald

**Affiliations:** National Institute of Standards and Technology, Gaithersburg, MD 20899, USA

**Keywords:** Half-life, ionization chamber

## Abstract

For over 30 years scientists in the NIST radioactivity group have been using their pressurized ionization chamber “A” (PIC “A”) to make measurements of radioactivity and radioactive half-lives. We now have evidence that some of those reported measurements were incorrect due to slippage of the source positioning ring over time. The temporal change in the holder caused an error in the source-height within the chamber, which was thought to be invariant. This unaccounted-for height change caused a change in the detector response and thus a relative error in measured activity on the order of 10^−5^ to 10^−3^ per year, depending on the radionuclide. The drifting detector response affected calibration factors and half-life determinations. After discovering the problem, we carried out historic research and new sensitivity tests. As a result, we have created a quantitative model of the effect and have used that model to estimate corrections to some of the past measurement results from PIC “A”. In this paper we report the details and results of that model. Meanwhile, we have fixed the positioning ring and are recalibrating the detector using primary measurement methods and enhanced quality control measures.

## 1. Introduction

Since the 1960’s the NIST pressurized ionization chamber “A” (PIC “A”) has been used for two main purposes. First, it has been used to maintain radioactivity standards through calibration factors for over 70 γ-ray emitting radionuclides. Most of those calibration factors were determined in the late 1970’s and some are still used for radioactivity determinations. Second, PIC “A” has been used to measure half-lives for over 50 γ-ray emitting radionuclides.

We now have evidence that some of those reported activity and half-life values were incorrect due to slippage of the sample positioning ring over time The temporal change in the holder caused an error in the source-height within the chamber, which was thought to be invariant. This unaccounted-for height change caused a change in the detector response and thus a relative error in measured activity on the order of 10^−5^ to 10^−3^ per year, depending on the radionuclide. The drifting detector response affected calibration factors and half-life determinations. After discovering the problem, we carried out historic research and new sensitivity tests. As a result, we have created a quantitative model of the effect and have used that model to estimate corrections to some of the past measurement results from PIC “A”. Meanwhile, we have fixed the positioning ring and are recalibrating the detector using primary measurement methods and enhanced quality control measures.

The route from investigation to corrections went as follows. First, historic artifacts and drawings of the instrument were used to estimate the height of the positioning ring at various dates between 1968 and 2010. Second, the detector response was measured as a function of height in the chamber for various radionuclides. Third, those data were used to model the response change for any γ-ray-emitting radionuclide over any time-span. Various aspects of the model were then validated by measurements of other radionuclides at various heights, analysis of historic NIST equivalent-activity values in the International Reference System (SIR), analysis of NIST half-life fit residuals and comparison of corrected calibration factors with new calibration factors determined by primary standardization methods.

## 2. The Measurement System

The NIST PIC “A” is a type “TPA Mk. 2” re-entrant chamber with a “1 inch”(nominal 25 cm) inner diameter, a “1/32 inch” (nominal 0.8 mm) wall thickness containing “20 atm” (20.×10^5^ Pa) of argon [1]. The detector was obtained from the Chalk River Laboratories of the National Research Council of Canada and was a forerunner to the Centronic^1^ IG-11.

**Fig. 1:**
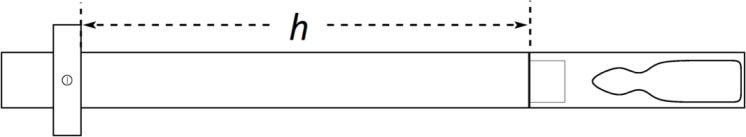
Sample holder, rotated 90 degrees counter-clockwise from its usual orientation. An ampoule sits in the bottom cup. The height, *h*, is shown.

The sources typically consist of aqueous solutions or gases contained within 5-mL glass ampoules that are placed inside a cylindrical sample-holder made of polymethyl methacrylate (acrylic). A brass positioning ring is fitted onto the holder a few cm from the top. The ring position on the holder is called *h*, as is shown in Fig. 1. When the holder is inserted into the chamber, this positioning ring rests on the shoulder of the chamber, and thereby sets the height of the sample within the chamber. The ring was thought to have been fixed by a set-screw in 1968. In January 2010, it was discovered that the set-screw did not drill into the holder, but only pushed against it, and was held in position by compression. The positioning ring has apparently been slipping an average of about 1 mm per year. Thus, the position of the sample in the chamber has moved deeper in the detector as a function of time. Since the detector response is sensitive to the height of the source, this slippage has caused a temporal change in the detector response for each radionuclide.

The standard procedures for using PIC “A” are described by Calhoun [2]. The methodology is similar to that used by Rytz [3]. The measurand is the ratio, *R*, of the detector response for a given radioactive source to that of a NIST radium-226 reference source. The calibration factor, *K*, (we call it the *“K*-value”), for a source of known activity, *A*, is defined as,

(1) K=A/R.

The use of the ratio to radium mitigates uncertainties caused by variations in the electrometer response over time. However, since the height sensitivity of the detector is dependent on the energy of the emitted γ-rays, the change in height will not necessarily affect the source of interest and the radium reference source by the same amount. That is, use of the ratio will not eliminate the effect of height-change on measured activity. Therefore, to quantify the effect of the positioning-ring slippage, in addition to gleaning *h* as a function of time, the variation of *R* as a function of *h* for each radionuclide needed to be determined.

## 3. The Model

### 3.1 Height Variation

**Fig. 2:**
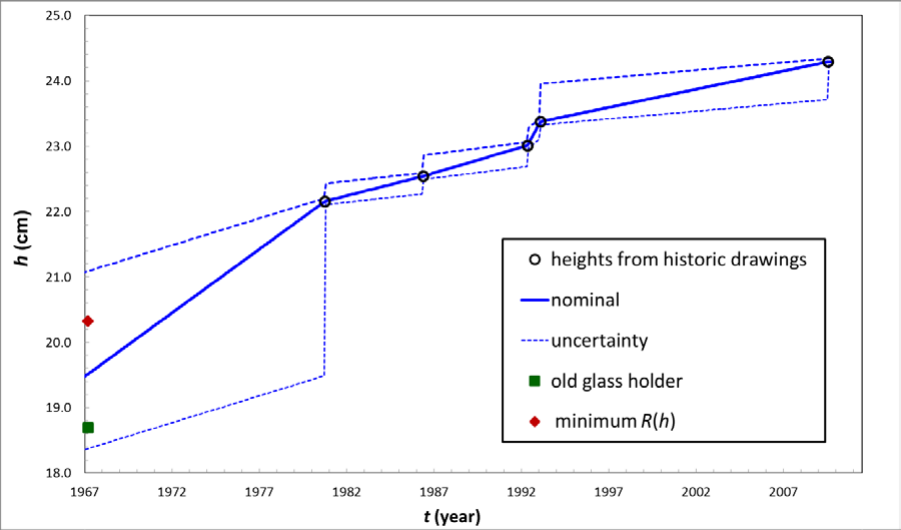
The solid line represents the assumed sample-holder height vs. time. The dashed lines represent the standard uncertainty, based on a uniform rectangular distribution. Two possible values for 1967 are described in the text, Sec. 3.1.

The height of the source positioning ring over time, *h*(*t*), was determined by studying historical drawings of the instrument from 1981, 1986, 1992, 1993 and 2010. No drawing of the sample holder from 1968, when the positioning-ring was set, has been found. One assumption is that the holder was made to be at the same height as the pre-1968 glass holder, which still exists. A second assumption is that the holder was made to be at the height which minimizes the slope of *R*(*h*) for a medium-energy γ-ray emitter like ^57^Co. An average of these two assumptions was chosen for the model. This assumption was consistent with various checks, described in Sec 3.3. Note that most calibration factors and half-life data date from after 1976, so the height at earlier dates is not very important. These height assumptions are listed in Table 1 and the resulting *h*(*t*) and its uncertainty are shown in Fig. 2. As evident from the graph, the slope of *h*(*t*) is not constant. Presumably *h*(*t*) is monotonic, since during routine operation the positioning ring would only feel forces that would tend to either increase *h* or leave it constant. Since the functional form of *h*(*t*) was unknown for times between the tabulated values, the uncertainty in *h* was estimated from a rectangular distribution (equal ignorance over the interval) between those measured values. The standard uncertainties^2^ are shown by the dashed lines in Fig. 2 and correspond to those standard uncertainty intervals calculated from the equally-probable (uniform) intervals divided by $\sqrt{3}$.

To use a historic *K*-value for an activity determination, the source-holder as it is presently used is now raised by spacers to the *h* corresponding to the *t* at which the *K*-value was originally determined. Predicted activity corrections for radionuclides for which we do not have sources on-hand are calculated, as described in the rest of Sec. 3.

### 3.2 Relative Response Function

**Fig. 3:**
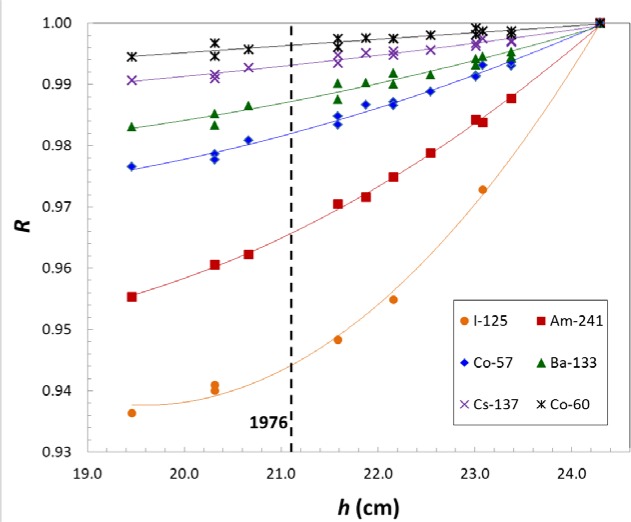
Graph of *R* versus height. The height corresponding to 1976 is shown by a dashed line. Quadratic fits for *R*(*h*) are shown by solid lines. The typical relative fit residual is 0.08 %.

The height dependence of the ionization current depends on the photon-emission spectrum for each radionuclide. In practice, the response is always measured as a ratio, *R*, to a ^226^Ra reference source. The height sensitivity of *R* is largest for radionuclides with γ-ray spectra dissimilar from ^226^Ra, particularly, emitters of low energy γ-rays and x-rays. The variation of *R* with *h* was initially measured experimentally using ^125^I, ^241^Am, ^57^Co, ^133^Ba, ^137^Cs and ^60^Co, as shown in Fig. 3. In order to use this data as a predictor of the effect for other radionuclides, we created a model of *R* as a function of both *h* and γ-ray energy. Using that model, *R* at *h* for a given radionuclide, *nuc*, which emits γ-rays of energy *E*_i_ with probability *P*_i_ detected with relative efficiency (current/photon), *ε*_i_, would be given by,

(2) $$R(nuc,h)=\frac{\sum_{i}R(E_{i},h)P_{i}\;\varepsilon_{i}}{\sum_{i^{\prime }}P_{i^{\prime }}\varepsilon_{i^{\prime }}}.$$

The *ε* curve was taken from the report by Calhoun, [2]. The ratio of *R* for a particular nuclide at two times would be given by,

(3) $$f=\frac{R(nuc,h(t_{1}))}{R(nuc,h(t_{2}){)}^{\prime }},$$

which can be expressed as,

(4) $$f=\frac{\sum_{i}R(E_{i,}h_{1})P_{i}\;\varepsilon_{i}}{\sum_{i^{\prime }}R(E_{i^{\prime }},h_{2})P_{i^{\prime }}\;\varepsilon_{i^{\prime }}}.$$

This *f* is the correction factor that would be used to adjust historic calibration factors from *t*_1_ to *t*_2_.

In order to determine *R*(*E*, *h*) from the data for the above-mentioned radionuclides (^125^I, ^241^Am, etc.), an effective energy was derived for each nuclide such that *R*(*nuc*,ℎ)=*R*(*E*_eff_,ℎ). This was done by iteration. The first approximation was to take the effective γ-ray energy for each nuclide as,

(5) $$E_{\text{eff}}(nuc)\approx \frac{\sum_{i}E_{i}P_{i}\;\varepsilon_{i}}{\sum_{i^{\prime }}P_{i^{\prime }}\varepsilon_{i^{\prime }}}.$$

The second iteration began by fitting the 2-dimensional function (*E*_eff_,ℎ), using the estimated *E*_eff_ values from the first iteration for each radionuclide. Then *f* was calculated for each radionuclide using Eqn. (4) with *t*_1_ = April 1981 and *t*_2_ = February 2010. Trial and error were then used to solve Eqn. (4) for a single energy *E*_eff_(*nuc*)=*E*_*i*_. A repeat of the second iteration showed that the *E*_eff_ values had converged to the values listed in Table 2. Note that these effective energies are appropriate for the calculation of *f*, but are not the same as the effective energies that would be used to characterize the detector efficiency.

**Fig. 4:**
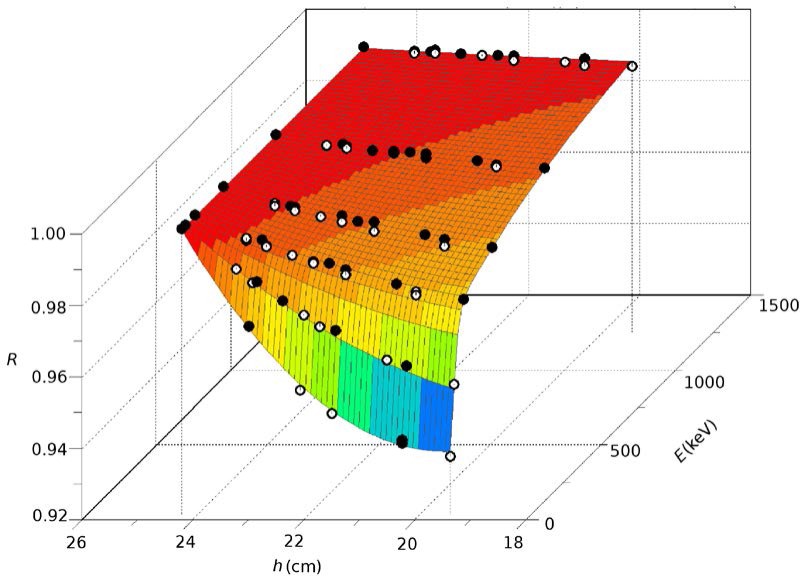
Parameterization of *R*(*E*,*h*) with RMS residual of 0.08 %.

Once *E*_eff_ were fixed, a least-squares fit of *R*(*E,h*), was performed using a function that was quadratic in *h* with both exponential and power terms in *E*. The function (Fig. 4) contains 8 parameters and was fit to 82 data points. To avoid edge effects, the fit used data with domains of *h* and *E* that exceeded those that would be used for calculating activity correction factors. The root mean square (RMS) relative fit residual was 0.08 %, which was about the same as the standard deviation of repeated *R* measurements for a given height.

The effect due to bremsstrahlung was parameterized using a Monte Carlo model of ^32^P and ^90^Y, and confirmed by ^85^Kr measurements. For a ^85^Kr source moved between *h* = 21.9 cm and *h* = 24.3 cm (corresponding to October 1979 and February 2010 in the model), we calculate *f* = (0.982 ± 0.031). The measured value for the two heights is *f* = (0.985 ± 0.015), in good agreement with the calculation. About half of the height effect on *R* for ^85^Kr is due to bremsstrahlung. For the other radionuclides considered here, bremsstrahlung was not significant.

### 3.3 Model Validation

**Fig. 5:**
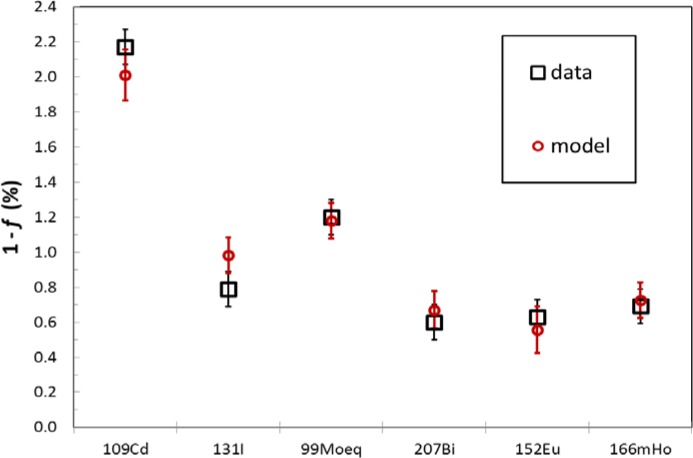
Test of the model of the correction due to moving the height of the source by 4.0 cm. The data uncertainty bars are standard deviations of repeated measurements, and the model uncertainties are based on the PIC “A” efficiency, *ε*, and the parameterization of *R*(*E*,*h*). Here “99Moeq” refers to ^99^Mo in equilibrium with ^99m^Tc.

The *R*(*E*,*h*) function was tested by making PIC “A” measurements with sources of ^109^Cd, ^131^I, ^99^Mo, ^207^Bi, ^152^Eu and ^166m^Ho. Setting *t*_1_ as the calibration date (mostly the late 1970’s) and *t*_2_ as the measurement date in 2010, the correction factors, *f*, differed from unity by between 0.6 % and 2.1 %. Measured values agreed well with the model, as shown in Fig. 5. Note that this test did not gauge the accuracy of *h*(*t*), since we merely chose two heights and both measured and calculated *f* as the source was moved between them.

Another test of the model was accomplished by comparing activity values determined by new primary measurements to those determined using corrected PIC “A” *K*-values. The three most stringent tests were ^67^Ga, ^99m^Tc and ^57^Co. After correcting the old *K*-values from c. 1977 to 2010, the old and new *K*-values differ on average by (0.4 ± 0.3 %), where the uncertainty is only the average uncertainty in the correction. This is a test of the complete model, *h*(*t*) and *R*(*E*,*h*).

**Fig. 6:**
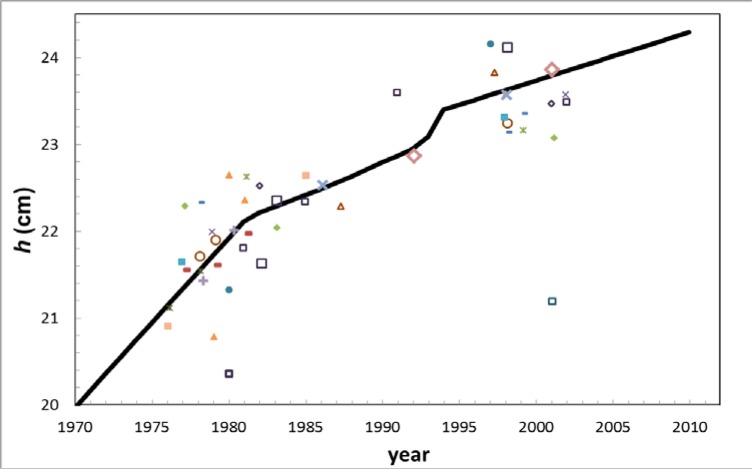
The *h*(*t*) model overlaid with SIR submissions by NBS/NIST for all radionuclides that have been submitted multiple times based on the same K-value, excluding ^59^Fe, which was very discrepant. The *R*(*nuc*,*h*) function was inverted to solve for the apparent change in height. Datasets for each nuclide were shifted vertically to overlay the model line. See text for uncertainty discussion.

Parts of the model were also compared with historic NIST/NBS submissions to the International Reference System (SIR). For various radionuclides, NIST had submitted numerous sources over the years to the SIR based on the same *K*-value. If we assume that the SIR ionization chamber did not suffer a similar problem to the one described here, then we can use the ratio of early and late NIST equivalent-activity values, *A*_e_, (similar to our *K*-values) as an indication of how PIC “A” has changed with time. To do this, we inverted *R*(*nuc*,*h*) to convert *A*_e_ into *h* for each radionuclide and then compared the implied height changes with our *h*(*t*) model. The SIR results for 19 radionuclides are shown in Fig. 6. As the SIR data only indicate the slope of *h*(*t*), not an absolute value, the height offset for each dataset (2 to 5 submissions for each radionuclide) was chosen so that the data straddle the model line. The slopes of the various datasets tend to agree with the model. One radionuclide, ^59^Fe, was excluded from this plot because it disagreed with the other data by 4 cm, probably for some reason other than this sample-holder effect. The uncertainties on the slopes are larger for high-energy γ-ray emitters and lower for low-energy γ-ray emitters, which are more sensitive to height. The scatter of the data in Fig. 6 indicates the uncertainty in this slope comparison.

**Fig. 7:**
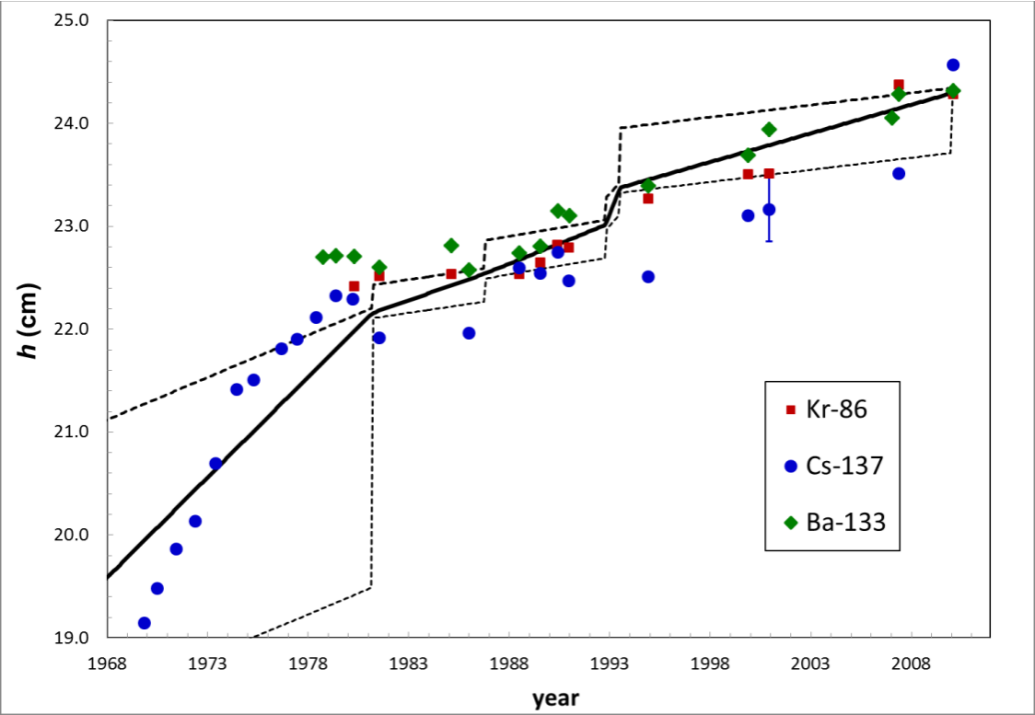
Half-life residuals confirming *h*(*t*) assumption. A typical uncertainty is shown for a ^137^Cs point. The scatter in the data also reflects the uncertainty. The data for each nuclide were shifted vertically to overlay the model line.

Another test was performed using half-live residuals. Although we later estimated half-life corrections, as presented in Sec. 4, we first used the existing data to check our model. We first calculated new world-average half-life values without NIST in the cohort. The data were taken from the Decay Data Evaluation Project [4]. We then fit the historic NIST data with the evaluated half-life and recorded the residuals from the fit. We then used the *R*(*nuc*, *h*) model to convert those residuals for *R* into residuals in *h*. Plotting these residuals versus time (Fig. 7) roughly confirmed the *h*(*t*) assumption beyond the 1981 height measurement. Recall that we set the 1968 value based on an assumption about what was done then. That assumed height is between the value from a linear extrapolation of measured heights and that suggested by these half-life residuals. Due to the extreme uncertainty of this assumption, we propose that any new half-life fits begin with 1981 data, ignoring data from before that time.

### 3.4 Results and Uncertainties for Calibration Factors

The uncertainties of the *f* calculations are usually mostly due to the *h*(*t*) interpolation. The standard uncertainties in *h*(*t*) are shown by the dashed lines in Fig. 2. This source of uncertainty caused asymmetric uncertainty in *f*. For ease of reporting, a symmetrized uncertainty was calculated to be the average of the plus and minus uncertainties. Due to the many assumptions underlying this analysis, there exists a large uncertainty in the uncertainty for these corrections. Multiplying the standard uncertainty by 2 is not a good estimate of a 95 % confidence level on the correction factor.

Table 3 shows an example for the result and uncertainty budget for ^155^Eu. In this case the *K*-value determination date was 1 January 1978, and the *K*-value usage date was 1 January 2001. Results for 11 radionuclides that are routinely measured in PIC “A” are listed in Table 4. They are tabulated by the relative change in response, *D* = 100(1 − *f*). The *t*_2_ value used for the calculations in Table 4 was February 2010, after the ring was fixed.

### 3.5 SIR Equivalence Values

The SIR equivalent activities, *A*_e_, are essentially calibration factors for the SIR ionization chamber [3], based on submissions from participating laboratories. For many radionuclides, NIST has repeatedly submitted samples to the SIR based on PIC “A” measurements. In those cases, the PIC “A” *K*-values were typically determined in the late 1970’s. Specifically, the more-recently reported NIST values have been higher than the actual activity. Thus, the NIST activity reported in those cases would be affected by the sample-holder drift. In Table 5 we present corrected *A*_e_ values and uncertainties for those cases, following the same methodology as employed for correcting *K*-values. We also list *u_D_* as the uncertainty in *A*_e_ due only to the height-correction, while *u_A_*_e_ is the total uncertainty in *A*_e_. Omitted from Table 5 are nuclides that have been superseded by more recent primary standardizations as well as the two cases in which the time duration was only 2 years between primary and secondary submission. Most of the changes in *A*_e_ have a magnitude similar to their combined standard uncertainties. However, we consider the uncertainty estimate for *f* to be much less robust than the uncertainty estimate for an activity determined by a primary measurement method. Therefore, these corrected activity values are meant to demonstrate the magnitude of the effect, but are not to be considered of acceptable quality to be included in any average leading to reference values.

There is no change to the NIST ^99m^Tc link to the SIR. In that case, the PIC “A” had been calibrated in March 2009 and used in May 2009 with the SIR Transfer Instrument. The model predicts 0.008 % correction for that case, which is insignificant compared to the activity uncertainty of 0.43 %. This was verified by checking the results from measuring the same ^57^Co sample before the PIC “A” calibration in 2009 and again in 2010. The results were the same with an uncertainty of 0.08 %.

## 4. Half-lives

### 4.1 Correction Method

For over 30 years, NIST has been reporting half-lives, *T_m_*, measured using PIC “A” (Unterweger, 2002). These half-lives were determined over various time spans from c. 1974 until 2001. The recently-discovered drift in the sample-positioning ring has affected these *T_m_* values. These discrepancies had been noted by other national laboratories and data compilers.

**Fig. 8:**
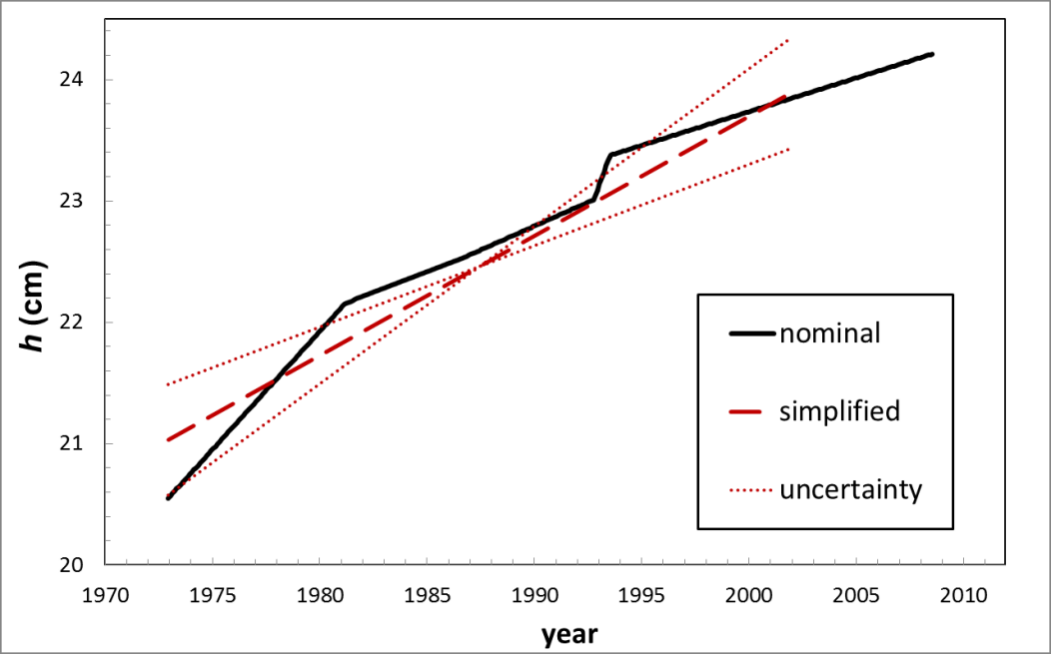
The solid line represents assumed model of sample-holder height vs. time. The dashed line represents the simplified, linear model used for these estimated half-life corrections and the dotted lines represent the uncertainty in the slope.

In this section we attempt to estimate the effect. To estimate the corrected half-life, *T*, we assume a linear change in *h* over time, as shown by the dashed line in Fig. 8.

Note that *T_m_* had been determined by Unterweger [5] assuming the usual decay law for a source producing signal *R*_0_ at time zero and signal *R* at time *t*:

(6) RR0=exp(−ln(2)tTm).

But in reality, due to the shifting positioning-ring, the measured decay is better approximated by,

(7) RR0=exp(−ln(2)tT)(1+ξt).

Where the slope ξ = *dR*/*dt* was determined using the model of *R*(*nuc,h*) for each radionuclide and the linear *h*(*t*) shown in Fig. 8. By equating Eqns. (6) and (7), one can solve for the relative change in apparent half-life for a 2-point half-life determination,

(8) $$z=\frac{T_{m}-T}{T_{m}}=\frac{T\ln(1+\xi t)}{\ln(2)t}.$$

For all cases reported here ξ*t* < 0.01, so to good approximation,

(9) $$z=\frac{\xi \;T_{m}}{\ln(2)}.$$

Thus, the measured half-lives can be corrected using *z* from Eqn. (9) as follows,

(10) $$T=T_{m}(1-z).$$

We can also express *T* in terms of the change in half-life, Δ,

(11) $$T=T_{m}-\Delta ,$$

(12) $$\Delta =\frac{\xi \;T_{m}^{2}}{\ln(2)}.$$

The uncertainty in *T* due to *T_m_* and ξ is given by,

(13) $$u_{T}=\sqrt{u_{T_{m}}^{2}+{\left(\frac{T_{m}^{2}}{\ln(2)}\right)}^{2}u_{\xi }^{2},}$$

where $u_{T_{m}}$ is the uncertainty reported by (Unterweger, 2002).

A relevant metric is the change in half-life relative to $u_{T_{m}}$,

(14) $$j=\frac{\Delta }{u_{T_{m}}}.$$

**Fig. 9:**
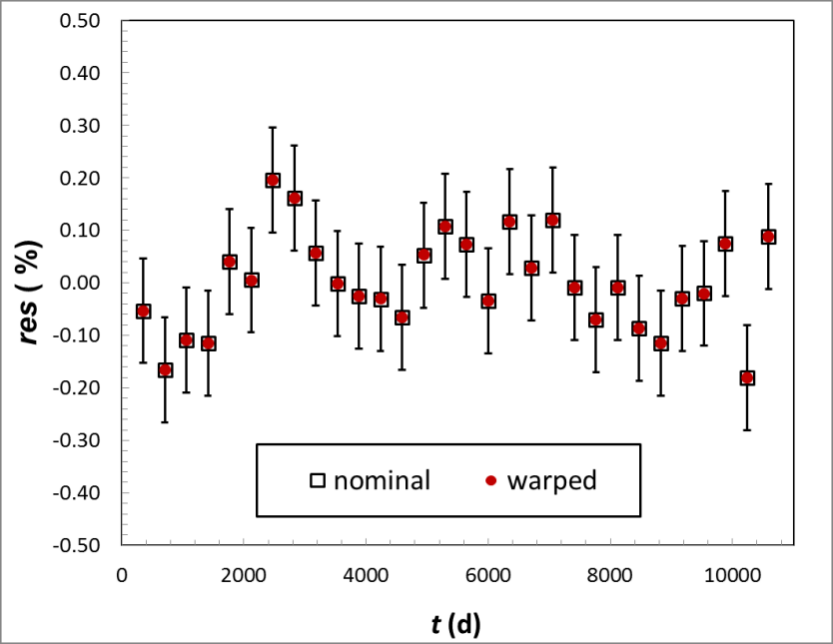
Relative residuals from least squares fit to simulated, ideal ^137^Cs decay data from Eqn. (6) (open squares) and warped data from Eqn. (7) (filled circles). The same Gaussian noise, the average magnitude of which is represented by the uncertainty bars, was applied to both data sets and used in the weighting of the fit. The fit value for half-life in the two cases differed by Δ = 109 d. In both cases the uncertainty returned was 9.5 d, χ^2^ per degree of freedom was 1, and no trend was visible in the residuals.

This derivation was based on a 2-point half-life determination. We tested the applicability of this approximation to the *T_m_* data, which were determined from a least-squares fit to multiple points of a decaying-source. We tested this approximation for ^137^Cs, which has a large *T*, large *j* and was measured for *t* = 0.9*T* and also tested it for ^155^Eu, which has a large ξ medium *j* and *t* =4*T*. We first simulated 30 decay-data points for the ideal case, using Eqn. (6) over the time-span *t*. We included Gaussian noise such that the uncertainty from a least-squares fit to the simulated data was $u_{T_{m}}$. We then simulated data using Eqn. (7) with the same noise as before, and calculated Δ from the respective fit parameters, *T_m_* and *T* It is important to note that the *χ*^2^ value and fit uncertainty were identical with and without the linear effect and no trend was visible in any of the residuals, shown in Fig. 9. Therefore, this effect would not have been detected from the original residuals. For ^137^Cs, the value from [5] is *T_m_* = (11018.3 ± 9.5 d). The simulation gave Δ = 109 d, whereas Eqn. (7) gave Δ = (104 ± 54) d. Likewise for ^155^Eu, *T_m_* = (1739.1 ± 0.5 d), the simulation gave Δ = 8.4 d and Eqn. (7) gave Δ = (8.5 ± 3.5) d. Those uncertainties are due to ξ = *dR*/*dt* only. Based on these two tests, I am satisfied that the approximations of Eqns. (7) through (9) are justified.

### 4.2 Corrected Half-lives

The results for all of the reported half-lives of [5] except ^3^H, which had been determined by gas counting, are listed in Table 6. For each nuclide, we list *T_m_* and $u_{T_{m}}$ from [5] the half-life shift Δ and corrected values *T* and *u_T_* calculated here, using Eqns. (11) and (13) respectively. Also tabulated are *j*, the change in half-life relative to $u_{T_{m}}$ and the ratio of the new to old uncertainty. Of the 63 half-life corrections reported here, 18 have *j* > 1 and are indicated in bold face in Table 6. Long-lived radionuclides, especially those that are low-energy photon emitters (^85^Kr, ^155^Eu), tend to have the largest relative corrections. In most cases the change in half-life and its uncertainty are small. That is, in most cases *j* < 1 and $u_{T_{m}}\approx u_{T}$. The uncertainty component due to d*R/*d*t* results from, in most cases, the estimate of the height of the positioning ring versus time. For radionuclides that emit γ-rays of various energies, the calculation of the ionization-chamber efficiency as a function of energy can contribute significantly to the overall uncertainty. Example uncertainty budgets for ^137^Cs and ^155^Eu are shown in Table 7.

**Fig. 10:**
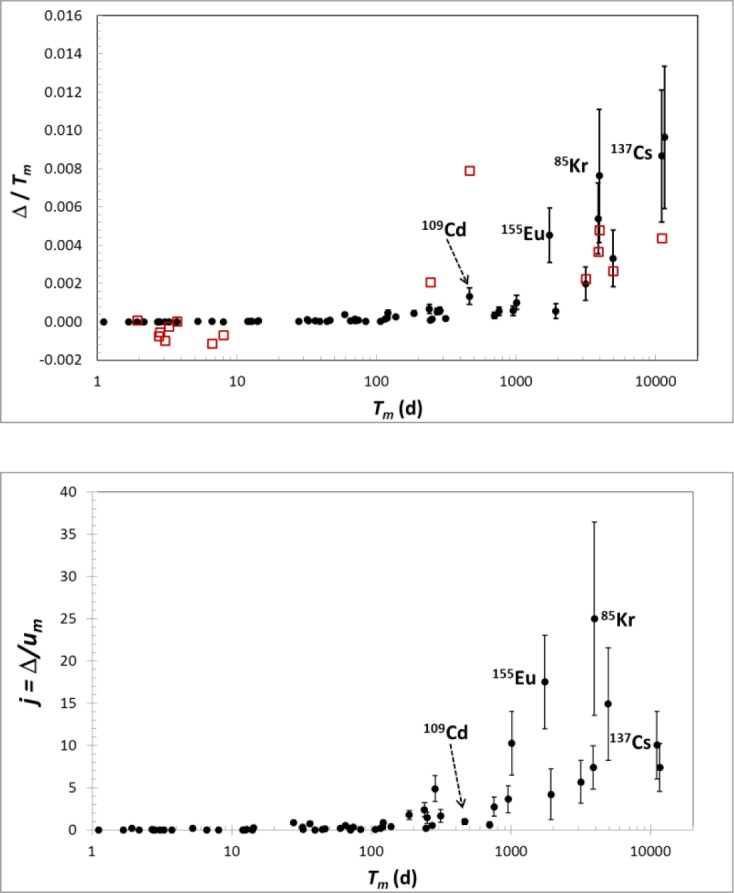
**Top:** Relative half-life correction. Closed circles are Δ with uncertainties described in text. Open squares are differences of Schrader [7] from Unterweger [5]. **Bottom:** Half-life corrections relative to original uncertainties [5]. The uncertainty bars are due to d*R*/d*t* only.

The top graph in Fig. 10 shows the relative change in half-life, Δ/*T_m_*, versus *T_m_*. The bottom graph in Fig. 10 shows $j=\Delta /u_{T_{m}}$. versus half-life. Also shown in the top plot are the differences of Schrader [7] measurements from Unterweger [5], (again relative to $u_{T_{m}}$), for comparison. This effect may account for the historic difference of Unterweger and Schrader for some long-lived radionuclides (e.g., ^85^Kr).

I consider these half-life corrections and their uncertainties to be rough approximations for use in identifying which half-lives are significantly affected, and at approximately what magnitude. For short half-lives (less than about 10 d), the calculated corrections are usually much smaller than the originally-stated uncertainties. A more accurate and more precise correction could be obtained by re-fitting corrected *R* data, restricting the dataset to dates beyond 1981, when the first known measurements of *h* exist. That method would avoid using the linear *h*(*t*) assumption, with its large uncertainty, as was done here. Such an approach would require the start and stop time for each half-life measurement.

## Figures and Tables

**Table 1:** Assumed values for the height of the positioning-ring on specific dates.

Date	*h* (cm)	*uh* (cm)
1/19/2010	24.29	0.05
7/28/1993	23.38	0.05
11/4/1992	23.01	0.05
11/16/1986	22.54	0.05
4/1/1981	22.16	0.05
1/1/1968	19.6	1.1

**Table 2:** Effective energies, *E*_eff_, for each given radionuclide, *nuc*.

*nuc*	*E*_eff_ (keV)
^125^I	33.6
^241^Am	59.6
^57^Co	124.0
^133^Ba	315.0
^137^Cs	662.
^60^Co	1251.

**Table 3:** Model results and uncertainty budget for ^155^Eu Activity correction factor, *f*, for height change from 1978 to 2001.

Component		*u*_i_		Comments
*h*(*t*)		0.00655		From rectangular distribution uncertainty for height as a function of date.
*r*(E,*h*) γ		0.00020		From residuals of fit.
*r*(*E,h*) γ		0.00098		From tests of fit.
*ε*		0.00117		From uncertainty in relative shape of efficiency curve for γ’s.
*r*(*h*) β		0.00001		From 20 % uncertainty in *R* vs. height for β’s.
*r*(*E*) β		0.00003		From 60 % uncertainty in shape of efficiency curve for β’s.
***u*_c_**		**0.0067**		**combined uncertainty of *f***
**Symmetrized, and corresponding asymmetric uncertainty component due to *h(t)***				
*f*	±		+	−
0.9836	0.0066		0.0033	0.0104
**Fractional contributions to *f***				
Β	0.0020			
γ	0.9980			

**Table 4:** Calculated K-value relative changes, Δ, for radionuclides commonly-measured in PIC “A”.

Nuclide	K-value date	Δ (%)	*u*_Δ_ (%)
^201^Tl	7/15/1976	2.5	0.7
^123^I	4/28/1976	1.5	0.4
^67^Ga	10/7/1977	1.5	0.5
^133^Xe	7/27/1988	1.40	0.15
^111^In	1/8/1977	1.4	0.4
^99^Mo	3/1/1977	1.3	0.4
^131^I	5/22/1978	1.0	0.4
^133^Ba	8/1/1983	0.97	0.11
^54^Mn	2/8/1979	0.45	0.20
^88^Y	9/1/1980	0.18	0.13
^99m^Tc	3/4/2009	0.03	0.19

**Table 5:** Reported NIST SIR equivalent activity, *A_e_*, corrected value, *A_e_*′, and relative difference, *D*. For each radionuclide, PIC “A” was calibrated at time *t*_1_ and used for the SIR submission at *t*_2_.

Nuclide	*t*_1_	*t*_2_	*A_e_* (MBq)	$u_{A_{e}}\left(\%\right)$	*A_e_*′ (MBq)	$u_{A_{e^{\prime }}}$ (%)	*D* (%)	*u_D_* (%)
^203^Hg	1976	1985	68.00	0.35	67.65	0.46	0.40	0.28
^99^Mo	1978	1998	64.69	0.24	64.13	0.36	0.80	0.29
^111^In	1977	1998	43.45	0.12	43.00	0.23	0.93	0.31
^131^I	1979	1998	40.50	0.14	40.23	0.21	0.62	0.26
^51^Cr	1981	1999	0.4893	0.00	0.4863	0.00	0.61	0.28
^137^Cs	1982	2001	27.63	0.10	27.54	0.10	0.36	0.12
^85^Sr	1977	2001	30.09	0.09	29.88	0.13	0.61	0.21
^59^Fe	1979	2001	14.64	0.06	14.61	0.06	0.19	0.12
^54^Mn	1979	2002	19.27	0.05	19.20	0.07	0.33	0.15
^88^Y	1980	2002	6.913	0.02	6.904	0.02	0.13	0.11
^133^Xe	1992	2001	896.10	3.70	889.59	4.49	0.73	0.28
^139^Ce	1987	1997	0.1344	0.00	0.1337	0.00	0.54	0.20
^153^Gd	1989	1998	369.4	2.50	366.5	2.77	0.79	0.32

**Table 6:** Measured half-lives from Unterweger [5] and corrected half-lives, *T*, from present work. For each radionuclide, the same unit is used for all non-ratio quantities. Radionuclides for which the correction is larger than the original uncertainty [5] are in **bold** face.

Nuclide	*T_m_*	$u_{T_{m}}$	Unit	*T*	*u_T_*	Δ	$j=\Delta /u_{T_{m}}$	$u_{T}/u_{T_{m}}$
^18^F	1.8295	0.0003	h	1.8295	0.0003	2E-07	0.0004	1.0
**^22^Na**	**950.97**	**0.15**	**d**	**950.4**	**0.4**	**0.6**	**4**	**2.4**
^24^Na	14.951	0.003	h	14.951	0.003	1E-06	0.0003	1.0
^32^P	14.263	0.003	d	14.262	0.003	0.001	0.3	1.0
^46^Sc	83.83	0.07	d	83.828	0.066	0.003	0.05	1.0
^51^Cr	27.7010	0.0012	d	27.6999	0.0013	0.0011	0.9	1.1
**^54^Mn**	**312.03**	**0.03**	**d**	**311.97**	**0.05**	**0.06**	**1.8**	**1.4**
^57^Co	272.11	0.26	d	271.95	0.27	0.16	0.6	1.0
^58^Co	70.77	0.11	d	70.77	0.11	0.004	0.03	1.0
^59^Fe	44.507	0.007	d	44.507	0.007	0.001	0.10	1.0
**^60^Co**	**1925.20**	**0.25**	**d**	**1924.0**	**0.9**	**1.2**	**5**	**3.7**
^62^Cu	9.673	0.008	m	9.672	0.008	7E-08	0.00001	1.0
^65^Zn	244.16	0.10	d	244.14	0.10	0.02	0.2	1.0
^67^Ga	3.2615	0.0005	d	3.2615	0.0005	2E-05	0.04	1.0
^75^Se	119.81	0.07	d	119.78	0.07	0.03	0.4	1.0
**^85^Kr**	**3935.7**	**1.2**	**d**	**3905**	**19**	**31**	**26**	**16**
^85^Sr	64.853	0.008	d	64.848	0.008	0.005	0.6	1.0
^88^Y	106.63	0.04	d	106.62	0.04	0.003	0.07	1.0
^99^Mo	65.924	0.006	h	65.924	0.006	0.0002	0.03	1.1
^99m^Tc	6.0072	0.0009	h	6.0072	0.0009	3E-06	0.004	1.0
^99A^Tc	6.012	0.003	h	6.012	0.003	3E-06	0.001	1.0
^103^Ru	39.31	0.04	d	39.31	0.04	0.0017	0.04	1.0
**^109^Cd**	**463.3**	**0.6**	**d**	**462.6**	**0.7**	**0.7**	**1.1**	**1.1**
**^110m^Ag**	**249.950**	**0.024**	**d**	**249.91**	**0.03**	**0.04**	**1.6**	**1.4**
^111^In	2.8048	0.0005	d	2.8048	0.0005	1E-05	0.03	1.0
^113^Sn	115.08	0.08	d	115.06	0.08	0.018	0.22	1.0
^117m^Sn	14.00	0.05	d	14.00	0.05	0.0004	0.01	1.0
^123^I	13.2235	0.0019	h	13.2235	0.0019	1E-05	0.01	1.0
^125^I	59.49	0.13	d	59.47	0.13	0.02	0.2	1.0
**^125^Sb**	**1007.56**	**0.10**	**d**	**1006.5**	**0.6**	**1.1**	**11**	**5.6**
^127^Xe	36.345	0.003	d	36.342	0.003	0.002	0.8	1.1
^131^I	8.0197	0.0022	d	8.0196	0.0022	9E-05	0.04	1.0
^131m^Xe	11.934	0.021	d	11.934	0.021	0.0004	0.02	1.0
**^133^Ba**	**3854.7**	**2.8**	**d**	**3832.3**	**10.8**	**22.4**	**8**	**3.8**
^133^Xe	5.2475	0.0005	d	5.2474	0.0005	1E-04	0.2	1.0
**^134^Cs**	**753.88**	**0.15**	**d**	**753.43**	**0.28**	**0.4**	**3**	**1.9**
**^137^Cs**	**11018.3**	**9.50**	**d**	**10915**	**55**	**104**	**11**	**5.8**
^139^Ce	137.73	0.09	d	137.70	0.09	0.04	0.4	1.0
^140^Ba	12.7527	0.0023	d	12.7525	0.0023	0.0002	0.08	1.0
^140^La	40.293	0.012	h	40.293	0.012	2E-05	0.002	1.0
^141^Ce	32.510	0.024	d	32.508	0.024	0.0022	0.09	1.0
**^144^Ce**	**284.53**	**0.03**	**d**	**284.35**	**0.08**	**0.18**	**5**	**2.4**
**^152^Eu**	**4947.2**	**1.1**	**d**	**4929**	**10**	**18**	**16**	**8.8**
**^153^Gd**	**239.47**	**0.07**	**d**	**239.29**	**0.10**	**0.18**	**2.6**	**1.5**
^153^Sm	46.2853	0.0014	h	46.2850	0.0030	0.0003	0.2	2.2
**^154^Eu**	**3145.2**	**1.1**	**d**	**3138**	**4**	**7**	**6**	**3.5**
**^155^Eu**	**1739.1**	**0.5**	**d**	**1731**	**3**	**8**	**19**	**7.8**
^166^Ho	26.794	0.023	h	26.794	0.023	8E-05	0.004	1.0
^169^Yb	32.015	0.009	d	32.011	0.009	0.004	0.4	1.0
^177^Lu	6.640	0.010	d	6.640	0.010	0.0001	0.01	1.0
**^181^W**	**121.10**	**0.06**	**d**	**121.03**	**0.07**	**0.06**	**1.0**	**1.1**
^186^Re	89.25	0.07	h	89.25	0.07	0.0010	0.014	1.0
^188^Re	17.001	0.022	h	17.001	0.022	2E-5	0.001	1.0
^188^W	69.78	0.05	d	69.77	0.05	0.012	0.25	1.0
^192^Ir	73.81	0.02	d	73.802	0.019	0.008	0.4	1.0
**^195^Au**	**186.10**	**0.05**	**d**	**186.01**	**0.06**	**0.09**	**1.9**	**1.3**
^198^Au	2.69517	0.00021	d	2.69516	0.00021	9E-06	0.04	1.0
^201^Tl	3.0456	0.0015	d	3.0456	0.0015	3E-05	0.02	1.0
^202^Tl	12.47	0.08	d	12.47	0.08	0.0003	0.004	1.0
^203^Hg	46.619	0.027	d	46.615	0.027	0.004	0.14	1.0
^203^Pb	51.92	0.04	h	51.92	0.04	0.0003	0.007	1.0
**^207^Bi**	**11523**	**15**	**d**	**11403**	**61**	**120**	**8**	**4.1**
^228^Th	698.6	0.4	d	698.4	0.4	0.2	0.7	1.1

**Table 6:** Uncertainty budgets for corrected half-lives, *T*, for ^137^Cs and ^155^Eu. The designation “A or B” refers to the type of uncertainty evaluation, as described in Taylor and Kuyatt [6].

Source	Description	A or B	^137^Cs *u*_i_ (%)	^155^Eu *u*_i_ (%)
***T****_m_*	Uncertainty for *T_m_* reported by [5]	B	.086	0.026
***h*(*t*)**	Due to standard uncertainty in position change with time.	B	0.46	0.19
***R*(*E*,*h*)**	Due to standard uncertainty in *R*(*E*,*h*) for a single photon energy.	B	0.17	0.037
***ε***	Due to standard uncertainty in detector efficiency as a function of energy.	B	0	0.032
		***u*_c_ (%)**	**0.50**	**0.20**

## Footnotes

1 Certain commercial equipment, instruments, or materials are identified in this paper to foster understanding. Such identification does not imply recommendation or endorsement by the National Institute of Standards and Technology, nor does it imply that the materials or equipment identified are necessarily the best available for the purpose.

2 All uncertainties reported in this paper are combined standard uncertainties in the corresponding measurand, unless otherwise noted.

## References

[1] Sharpe J, Wade F (1953). A Re-entrant thimble ionisation chamber. Atomics.

[2] Calhoun JM (1986). Radioactivity Calibrations with the NBS “4π”γ Ionization Chamber, and Other NBS Radioactivity Calibration Capabilities, NBS SP 250-10.

[3] Rytz, A A (1978). International coherence of activity measurements. Environ Int.

[4] Bé MM Decay Data Evaluation Project (DDEP).

[5] Unterweger MP (2002). Half-life measurements at the National Institute of Standards and Technology. Appl Radiat Isot.

[6] Taylor BN, Kuyatt CE (1994). Guidelines For evaluating and Expressing the Uncertainty of NIST Measurement Results, NIST TN 1297.

[7] Schrader H (2004). Half-life measurements with ionization chambers - A study of systematic effects and results. Appl Radiat Isot.

